# Supplementary material for: Facemasks: A Looming Microplastic Crisis
Source: Int J Environ Res Public Health. 2021 Jul 1;18(13):7068. doi: 10.3390/ijerph18137068 (PMC8297027; doi:10.3390/ijerph18137068)
Supplement: Supplementary file 1 [file ijerph-18-07068-s001.zip › ijerph-1276880-supplementary.pdf]

Supplementary Document

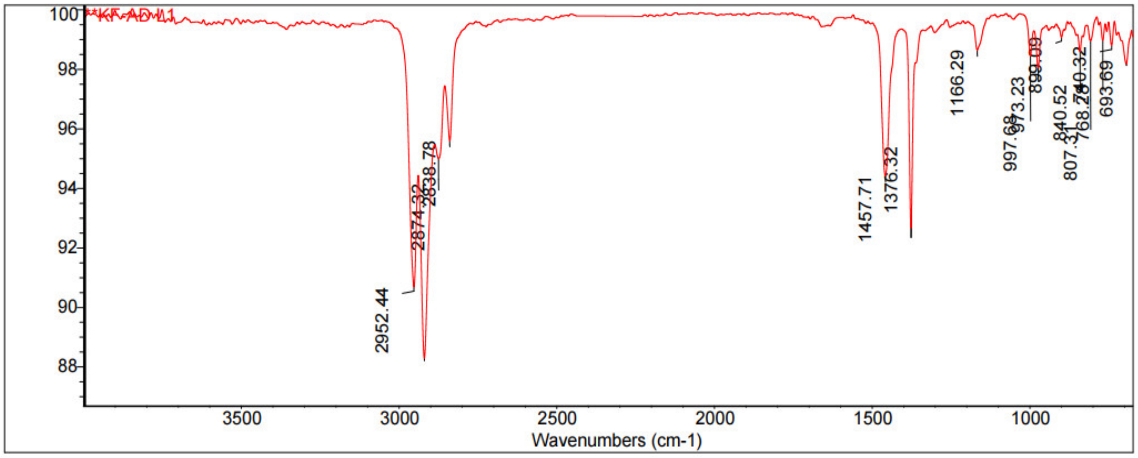

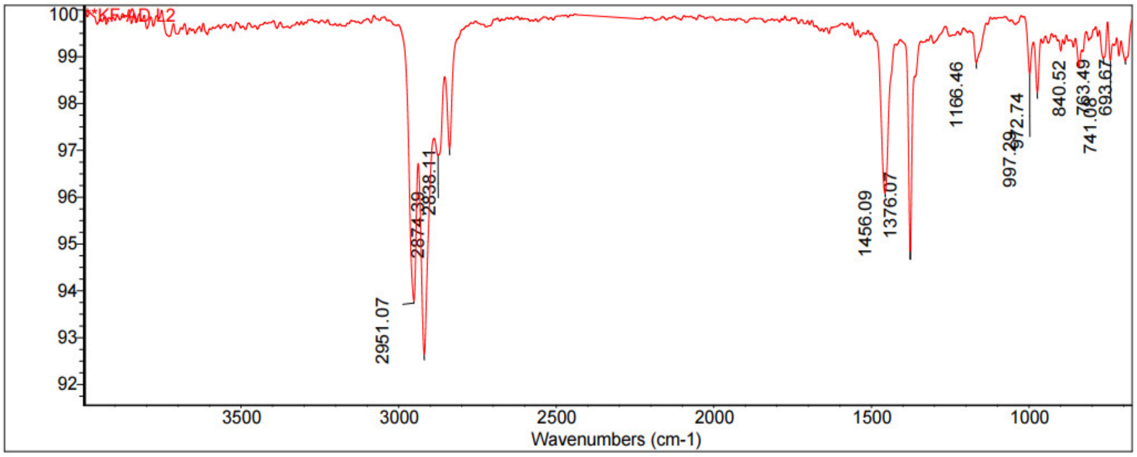

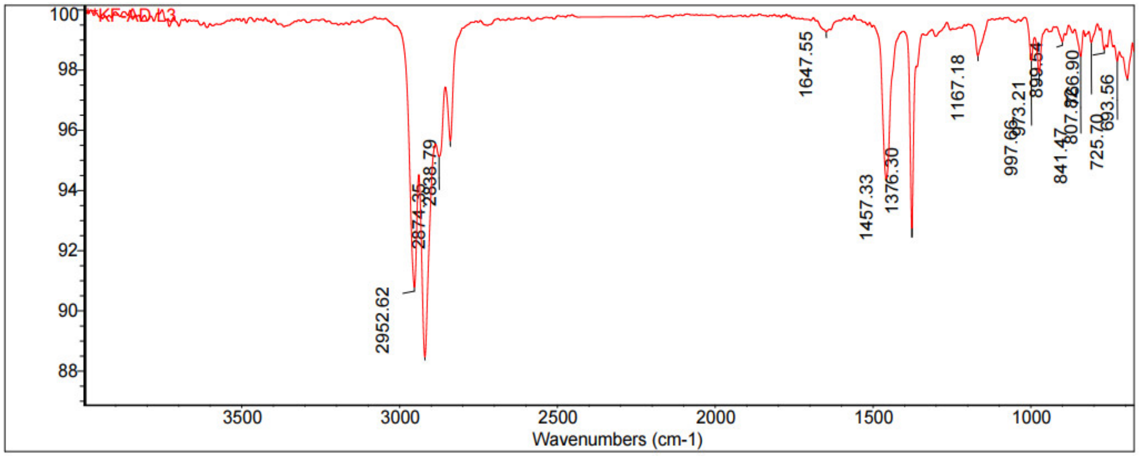

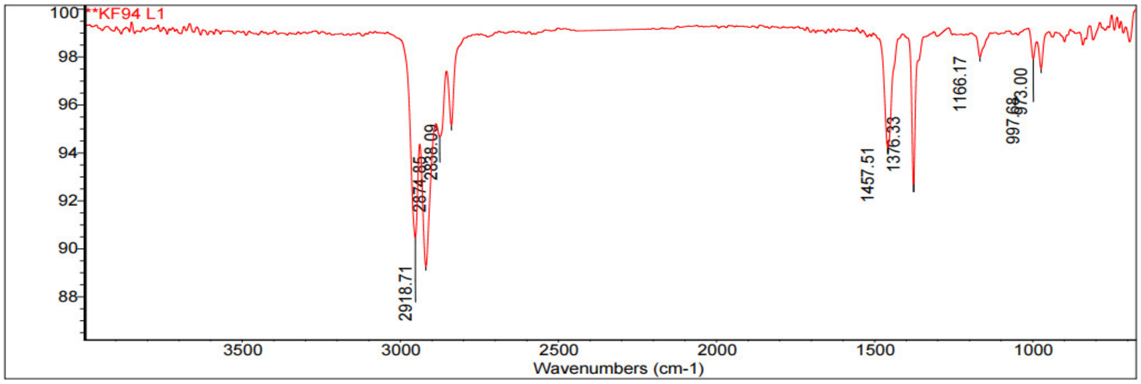
**KF94 L1
Wavenumbers (cm-1)
2918.71
2874.85
2838.09
1457.51
1376.33
1166.17
997.68
973.00

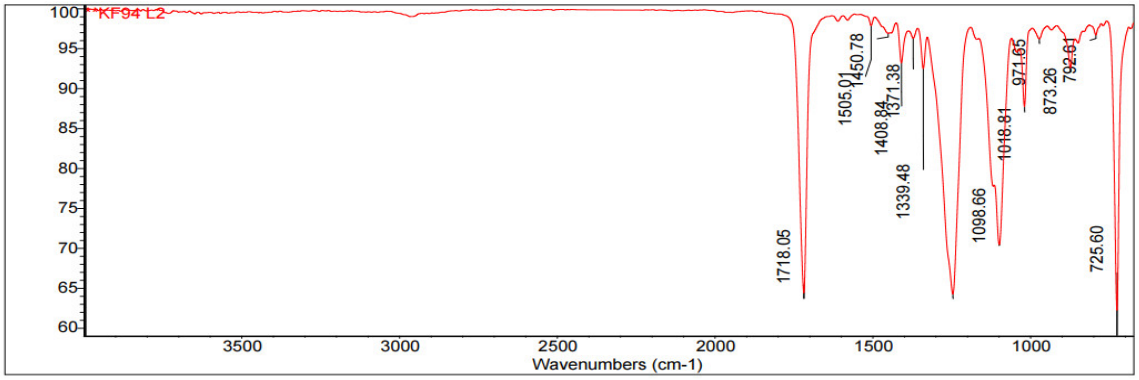
**KF94 L2
Wavenumbers (cm-1)
1718.05
1505.01
1450.78
1408.84
1371.38
1339.48
1098.66
1018.81
971.65
873.26
792.81
725.60

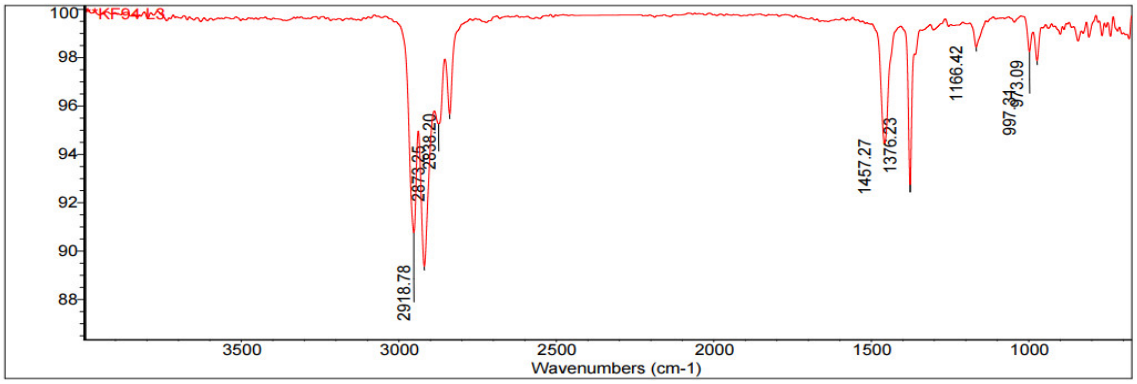
**KF94 L3
Wavenumbers (cm-1)
2918.78
2873.25
2838.20
1457.27
1376.23
1166.42
997.31
973.09

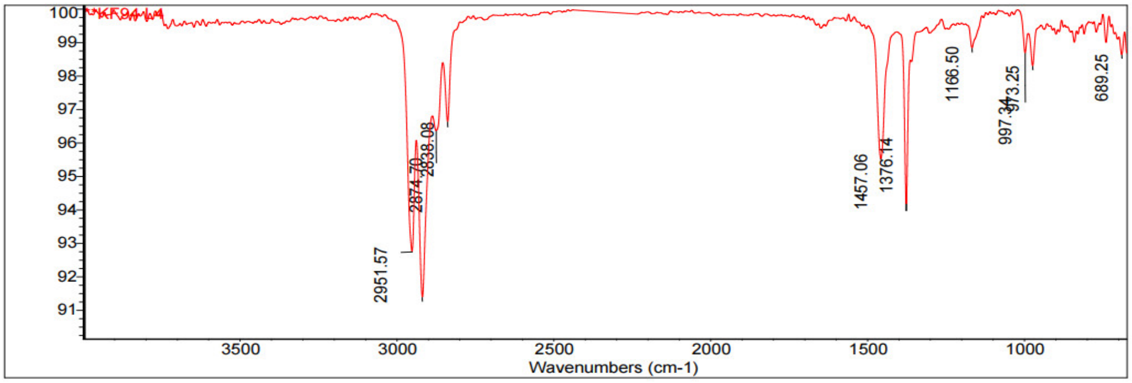
**KF94 L4
Wavenumbers (cm-1)
2951.57
2874.70
2838.08
1457.06
1376.14
1166.50
997.34
973.25
689.25

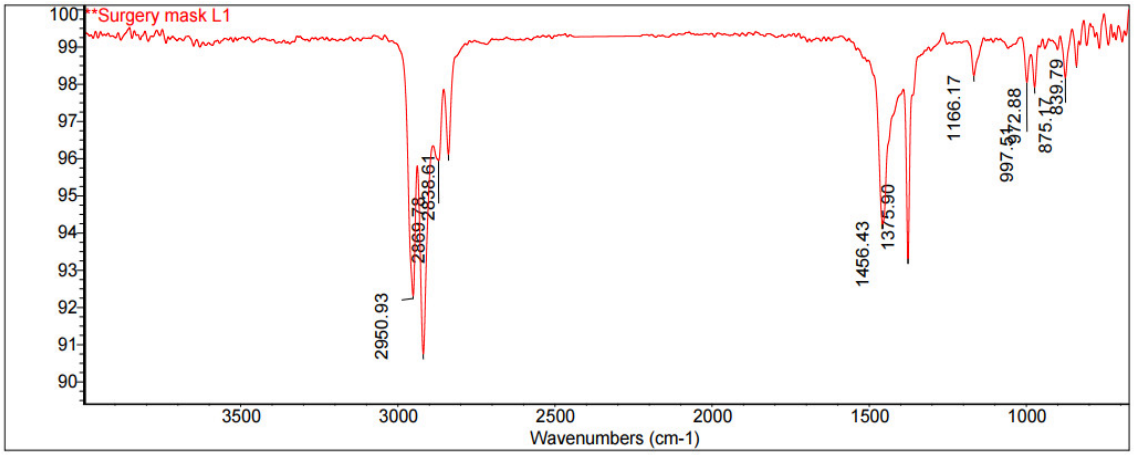
**Surgery mask L1
Wavenumbers (cm-1)
2950.93
2869.78
2838.61
1456.43
1375.90
1166.17
997.51
972.88
875.17
839.79

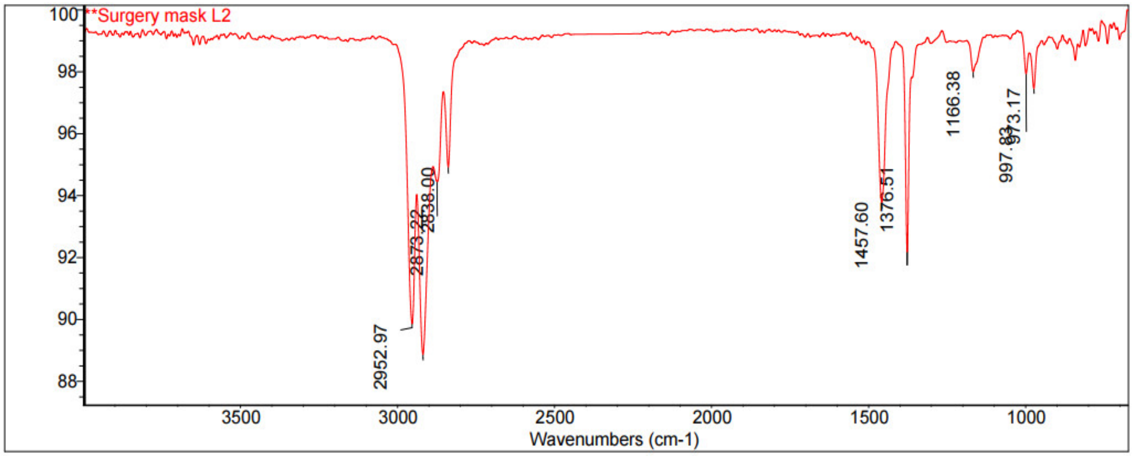
**Surgery mask L2
Wavenumbers (cm-1)
2952.97
2873.22
2838.00
1457.60
1376.51
1166.38
997.83
973.17

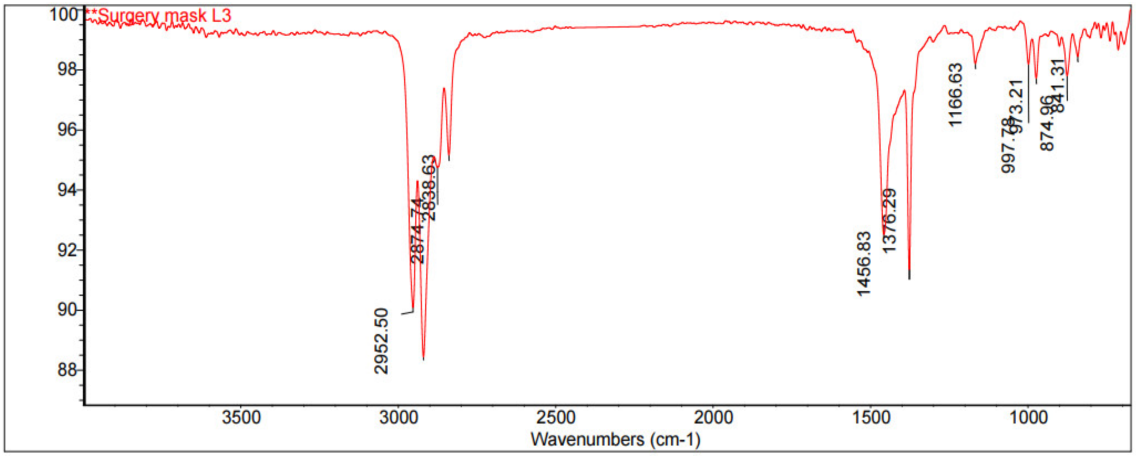
**Surgery mask L3
Wavenumbers (cm-1)
2952.50
2874.74
2838.63
1456.83
1376.29
1166.63
997.79
973.21
874.96
841.31

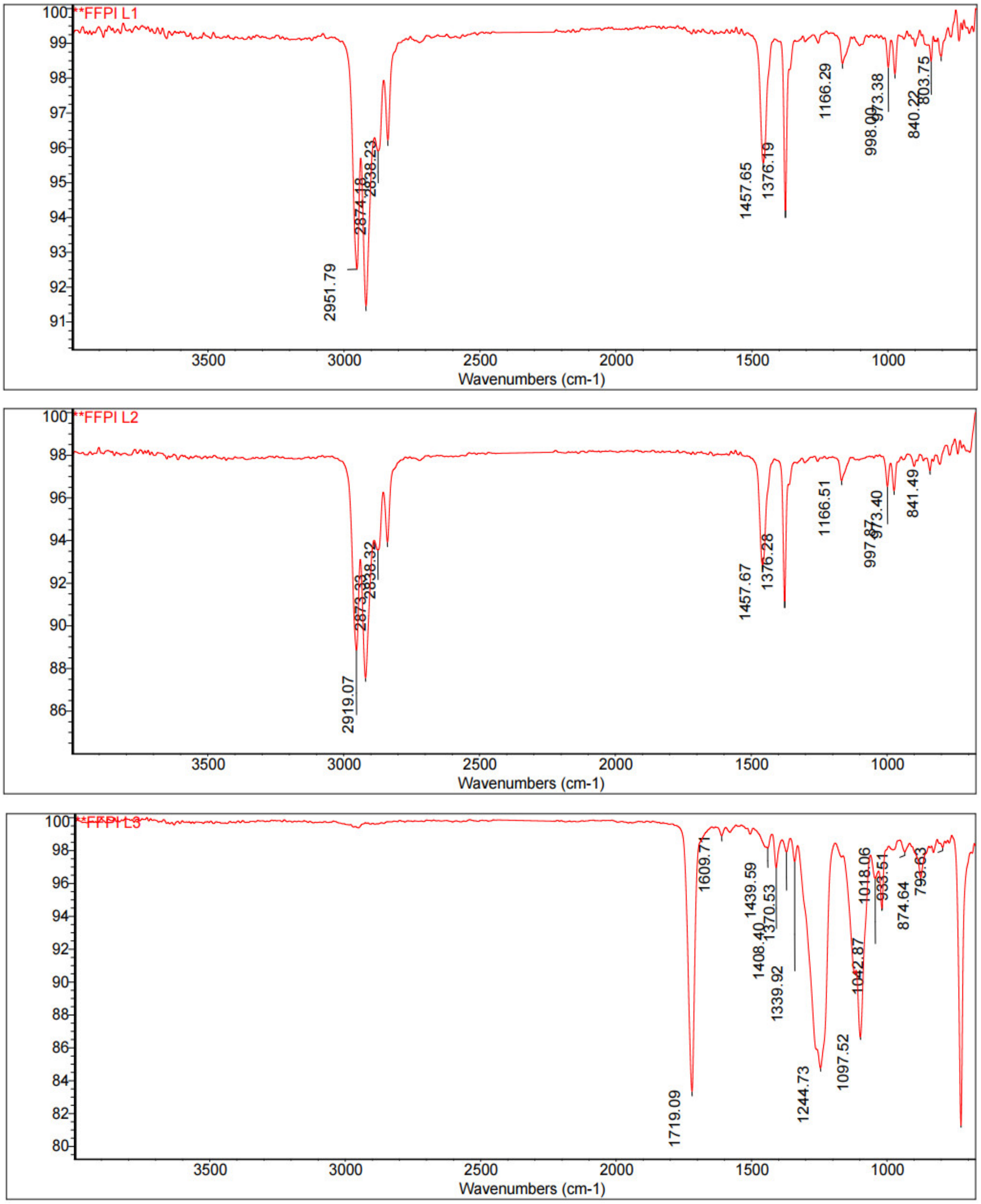

Figure S1 FTIR for all layers of each face mask (L1 refers to the outer layer of the mask and the layer number increase when it gets closer to the inner layer. i.e. the innermost layer (the layer touching the skin of an individual) of a FFP1 mask is L3)

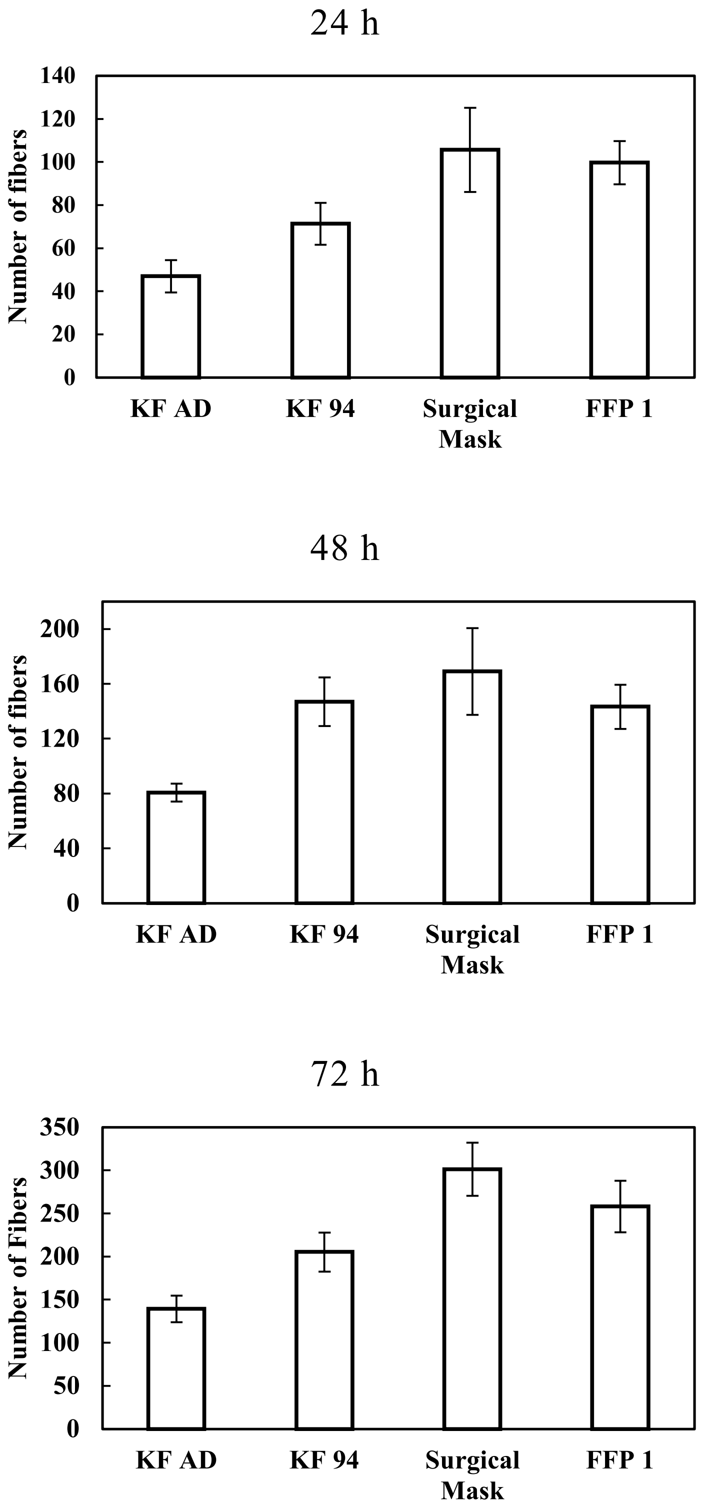

Figure S2. Number of retained microplastic fibers on the filter after shaking in MiliQ water at 150 rpm for 24, 48, and 72 h.
